# Boldo

**Published:** 1872-12

**Authors:** 


Boldo.—This is the name of a new remedy which has been recently introduced into Europe. It is imported from Chili, where it is distilled from the leaves of a tree, of the genus *Monimiacæ.* Its reputation appears to rest upon a pretty slender basis, and not upon the results of any trustworthy experiments. Thus far, it has been administered empirically for the more frequent affections of the liver. As in the case of *cundurango*, its use is most strongly recommended by charlatans, pecuniarily interested in its success, and like that drug, its popularity will probably be of very short duration.

